# Anesthesiological considerations and complications in robotic surgery: a retrospective analysis

**DOI:** 10.1007/s11701-026-04045-6

**Published:** 2026-10-12

**Authors:** P. Kuhn, M. Oremek, A. L. Kuenster, A. Labonte, M. Kroeplin, T. O. Vilz, J. Kalff, M. Soehle, M. Thudium

**Affiliations:** 1 Department of Anesthesiology and Intensive Care Medicine, University Hospital Bonn, Venusberg Campus 1, 53127 Bonn, Germany; 2 Department of Surgery, University Hospital Bonn, Bonn, Germany; 3 Department of Surgery, Campus Benjamin Franklin, Charité - Universitätsmedizin Berlin, Berlin, Germany; 4 Institute for Medical Biometry, Informatics, and Epidemiology, University of Bonn, University Hospital Bonn, Bonn, Germany

**Keywords:** Robotic surgery, Anesthesia, Complications, Fluid management, Surgical duration, Clavien-Dindo classification

## Abstract

Robotic-assisted surgery has become increasingly prevalent across various surgical disciplines. Understanding anesthesiological factors influencing perioperative outcome is essential for optimizing patient care. This retrospective analysis evaluated 200 patients who underwent robotic surgery across three surgical categories: gastrointestinal (GI, n=60), hepatopancreticobiliary (HPB, n=28), and colorectal (n=112) procedures. We conducted an exploratory analysis of associations between anesthesiological parameters—including fluid management, surgical duration, and blood loss—and clinical outcomes such as intensive care unit (ICU) length of stay, total hospital length of stay, complication severity (Clavien-Dindo classification) using univariable and multivariable regression models. The exploratory analyses suggested potential associations between surgical duration and outcomes across surgical specialties in multivariable analysis. For GI procedures, longer surgical duration was associated with higher odds of severe complications (Clavien-Dindo grade ≥III; OR = 1.80 (1.21, 2.66)). In colorectal surgery, surgical duration was independently associated with both development of severe complications (OR = 1.28 (1.01, 1.62)) and total hospital length of stay (log-scale, β = 0.08 (0.03, 0.12)). Fluid intake was associated with an increased hospital length of stay in colorectal surgery (log-scale, β = 0.10 (0.03, 0.17)). Anesthesiological management, particularly surgical duration and fluid management, showed associations with perioperative outcomes in robotic surgery. The estimated effects provide preliminary insights into the magnitude of these associations and may serve as a basis for hypothesis generation and the design of future prospective studies. Further studies are needed to confirm these findings and to investigate the potential clinical relevance of anesthesiological and perioperative management strategies.

**Supplementary Information:**

The online version contains supplementary material available at https://doi.org/10.1007/s11701-026-04045-6.

## Introduction

Robotic-assisted surgery has changed surgical practice across multiple disciplines, promising advantages such as enhanced visualization, improved dexterity, and reduced surgical trauma [1]. Robotic-assisted surgery systems have been increasingly adopted for complex gastrointestinal, hepatobiliary, and colorectal procedures. However, robotic surgery presents unique anesthesiological challenges, including prolonged operative times, steep Trendelenburg positioning, pneumoperitoneum, and limited intraoperative access to the patient [2, 3].

Anesthesiological management plays a crucial role in determining perioperative outcomes. Factors such as fluid administration, maintenance of hemodynamic stability, but also preoperative patient status can directly influence postoperative recovery, complication rates, and hospital length of stay. Despite the growing adoption of robotic techniques, comprehensive analyses examining the relationship between anesthesiological parameters and surgical outcomes remain limited [2].

Previous studies have established prolonged surgical duration as a risk factor for complications across different surgical specialities, yet its specific impact in the context of robotic procedures across different surgical specialties has not been fully elucidated [4]. Similarly, optimal fluid management strategies during robotic surgery remain a subject of ongoing investigation, with both restrictive and liberal approaches having potential advantages and disadvantages [5–7].

This retrospective analysis aimed to evaluate the relationship between key anesthesiological parameters—including intraoperative fluid management, surgical duration, and blood loss—and clinically relevant outcomes such as duration of ICU admission, total hospital length of stay, as well as frequency and severity of complications across three major robotic surgical categories. A further objective was to derive preliminary effect estimates that may inform the design and sample size planning of future retrospective studies. Given the exploratory nature and retrospective observational design of the study, the analyses were intended to identify potential associations and generate hypotheses rather than to establish causal relationships.

## Materials and methods

### Study design and patient population

This retrospective cohort study analyzed data from 200 consecutive patients who underwent robotic-assisted surgery at a single tertiary care center. To account for the respective particularities of the different operative procedures, patients were stratified into three surgical groups: gastrointestinal (GI, n=60), hepatobiliary (HPB, n=28), and colorectal (n=112). The study was conducted in accordance with institutional review board requirements (Ethics committee of the University of Bonn: 2025/370) and followed the principles of the Declaration of Helsinki.

### Surgical procedures

GI procedures included gastric and small bowel resections as well as esophageal resections. HPB procedures predominantly consisted of pancreaticoduodenectomy (Whipple procedures) and other complex hepatopancreaticobiliary resections. Colorectal procedures encompassed both benign and malignant pathologies requiring segmental or total colectomy as well as deep anterior rectum resections.

All procedures were performed using the da Vinci Surgical System (Intuitive Surgical, Sunnyvale, USA) by experienced surgeons who had completed the institutional training for robotic surgery.

### Data collection

Demographic and clinical data were extracted from electronic medical records and anesthesia information management systems. Variables collected included:

Patient characteristics: age, sex, body mass index (BMI), American Society of Anesthesiologists (ASA) physical status classification.

The following intervals were recorded for each patient: anesthesia duration, ventilation time, surgical duration, docking time.

Registered fluid management parameters were crystalloid administration, colloid administration, red blood cell transfusion (RBCs), fresh frozen plasma transfusion (FFPs), Platelet concentrate transfusion (TKs). Fluid intake was defined as the sum of crystalloid administration, colloid administration, RBC transfusion, FFP transfusion, and platelet concentrate transfusion. Fluid output was defined as the sum of intraoperative blood loss and urine output.

For outcome measures we investigated intraoperative blood loss (l), as documented by the surcigal/anesthesia team, ICU length of stay (number of days from ICU admission to ICU discharge), total hospital length of stay (number of days from hospital admission to hospital discharge), and postoperative complications classified according to the Clavien-Dindo classification [8].

The Clavien-Dindo classification system ranges from grade I (minor deviation from normal postoperative course) to grade V (death). For analytical purposes, complications were dichotomized into non-severe (grades I-II) and severe (grades III-V), with the latter requiring surgical, endoscopic, or radiological intervention, or resulting in life-threatening complications or death [8]. We chose this binary approach to outcome to clarify variables associated with more severe complications while at the same time compromising some of the classification’s ability to distinct between stages of severity.

### Statistical analysis

Continuous variables are presented as mean with standard deviation (SD) and as median with first (Q1) and third quartiles (Q3). Categorical variables are summarized as absolute and relative frequencies. Descriptive statistics were stratified by surgical procedure.

Univariable and multivariable linear regression analyses were performed separately for each surgical procedure (colorectal surgery, GI surgery and HPB surgery) to assess associations between anesthesiological parameters (ASA status, duration of surgery, fluid intake, fluid output) and continuous outcome variables, including intraoperative blood loss, ICU length of stay, and total hospital length of stay. As an additional sensitivity analysis, net fluid balance (intake minus output) was modeled in place of fluid intake and fluid output for the linear regression outcomes; results are presented in Supplementary Tables S4 , S5 and S6. In addition, univariable and multivariable logistic regression analyses were conducted within each surgical procedure to evaluate associations between ASA status, surgical duration, fluid intake, and fluid output and the binary outcome of postoperative complications as classified by the Clavien–Dindo system. For all regression models, heteroscedasticity-consistent standard errors were used to provide robust inference in the presence of potential heteroscedasticity and the relatively small sample size. Specifically, the HC3 estimator was applied to the estimated covariance matrix, providing a leverage-adjusted correction that is particularly suitable for small samples.

We specifically focused on this restricted set of independent variables to provide a meaningful model output: ASA status as a composite indicator of overall physical status and comorbidities, duration of surgery which includes procedure complexity, and fluid status as a central modifiable anesthesiological variable. Univariable analyses were performed to explore unadjusted associations between individual predictors and outcomes, while multivariable models were used to account for potential confounding and to estimate associations adjusted for clinically relevant covariates. Independent variables were prespecified a priori based on clinical relevance. Regression analyses were stratified by surgical category to account for procedure-specific characteristics. Thus, the results represent within-group associations and were not designed to compare complication rates between different surgical procedures.

In linear regression analyses, regression coefficients are reported with corresponding 95% confidence intervals. In logistic regression analyses, effect estimates are presented as odds ratios (OR) with 95% confidence intervals. Regression coefficients and odds ratios are reported per one-unit increase in the corresponding predictor (e.g., per additional hour of surgical duration, per additional liter of fluid intake or output), unless otherwise specified. ICU and total hospital length of stay were log-transformed prior to linear regression analysis.

All statistical analyses and figures were generated using R statistical software (version 4.4.3). All tests were two-sided, and a p-value < 0.05 was considered statistically significant. All analyses were exploratory in nature; therefore, p-values are reported for descriptive purposes only and no adjustment for multiple testing was applied. Missing data were not imputed, and all analyses were performed using complete cases for the variables included in the respective analysis. Consequently, the number of evaluable patients (valid n) may vary between analyses and is reported for each analysis.

Additionally, variance inflation factors (VIF) were assessed to evaluate potential multicollinearity among the covariates. VIF values remained below 1.5 for colorectal and GI procedures and below 5 for HPB procedures, indicating no substantial multicollinearity among the covariates included in the respective models. The multivariable logistic model for severe complications was not fitted for the HPB subgroup, since the minority outcome class comprised only 3 of 28 patients, precluding stable estimation.

## Results

A total of 200 patients was included in the analysis, with a median age of 62 years. Of these, 87 patients (43.5%) were female and 113 (56.5%) were male. Detailed descriptive statistics of patient-related characteristics, stratified by surgical procedure, are presented in Table 1, while operation-related characteristics are summarized in Table 2.

**Table 1:** Baseline demographic characteristics of the study population stratified by surgical subgroup (gastrointestinal [GI], hepatopancreatobiliary [HPB], and colorectal) and overall cohort. Continuous variables are presented as mean (standard deviation (SD)) and median [first quartile (Q1), third quartile (Q3)], and categorical variables are presented as absolute and relative frequencies; N denotes the total number of observations per group and n denotes the number of valid observations

		GI (N=60)	HPB (N=28)	Colorectal (N=112)	All (N=200)
**Age (years)**	Mean (SD)	64.7 (11.2)	64.8 (14.3)	55.2 (18.1)	59.4 (16.4)
	Median [Q1, Q3]	65.0 [59.0,73.0]	67.5 [55.3,76.3]	57.0 [42.5,68.3]	62.0 [51.0,73.0]
		n = 60	n = 28	n = 112	n = 200
**Sex**	Male	39 (65.0%)	17 (60.7%)	57 (50.9%)	113 (56.5%)
		n = 60	n = 28	n = 112	n = 200
**Height (cm)**	Mean (SD)	173.3 (9.6)	174.4 (8.6)	172.6 (9.3)	173.1 (9.27)
	Median [Q1, Q3]	173.0 [165.0,180.0]	173.5 [169.5,181.0]	173.0 [167.0,178.0]	173.0 [167.3,180.0]
		n = 57	n = 28	n = 105	n = 190
**Weight (kg)**	Mean (SD)	80.0 (20.5)	81.7 (20.7)	75.8 (17.9)	78.0 (19.2)
	Median [Q1, Q3]	75.0 [67.0,91.0]	77.5 [71.0,83.3]	74.0 [65.0,85.0]	75.0 [65.0,87.8]
		n = 57	n = 28	n = 105	n = 190
**BMI (kg/qm)**	Mean (SD)	26.6 (6.7)	26.7 (5.7)	25.3 (5.0)	25.9 (5.7)
	Median [Q1, Q3]	24.9 [22.9,28.0]	25.8 [24.0,29.0]	25.0 [22.00,28.1]	25.0 [22.4,28.1]
		n = 57	n = 28	n = 105	n = 190
**ASA**	I / II	31 (51.7%)	20 (71.4%)	81 (72.3%)	132 (66.0%)
	III	29 (48.3%)	8 (28.6%)	31 (27.7%)	68 (34.0%)
		n = 60	n = 28	n = 112	n = 200

**Table 2:** Operation-related characteristics of the study population stratified by surgical subgroup (gastrointestinal [GI], hepatopancreatobiliary [HPB], and colorectal) and overall cohort. Continuous variables are presented as mean (standard deviation (SD)) and median [first quartile (Q1), third quartile (Q3)], and categorical variables are presented as absolute and relative frequencies; N denotes the total number of observations per group and n denotes the number of valid observations

		GI (N=60)	HPB (N=28)	Colorectal (N=112)	All (N=200)
**Ventilation time (h)**	Mean (SD)	9.55 (2.58)	8.92 (2.14)	9.14 (2.29)	9.23 (2.36)
	Median [Q1, Q3]	9.60 [8.03,11.77]	9.00 [7.65,9.65]	9.13 [7.33,10.80]	9.30 [7.50,10.88]
		n = 59	n = 28	n = 107	n = 194
**Surgical duration (h)**	Mean (SD)	8.04 (2.31)	7.40 (2.01)	7.34 (2.17)	7.56 (2.21)
	Median [Q1, Q3]	8.00 [6.35,9.79]	7.65 [6.47,8.36]	7.19 [5.72,8.80]	7.60 [5.94,8.96]
		n = 59	n = 28	n = 108	n = 195
**Robotic assisted**	Mean (SD)	5.76 (2.32)	5.47 (3.67)	5.16 (2.03)	5.38 (2.23)
**part (h)**	Median [Q1, Q3]	5.73 [4.18,6.96]	4.10 [3.87,6.30]	4.87 [3.83,6.25]	4.98 [3.88,6.67]
		n = 47	n = 7	n = 85	n = 139
**Blood loss (l)**	Mean (SD)	0.31 (0.46)	0.66 (0.92)	0.11 (0.31)	0.25 (0.52)
	Median [Q1, Q3]	0.10 [0.00,0.50]	0.30 [0.07,0.72]	0.00 [0.00,0.10]	0.00 [0.00,0.21]
		n = 59	n = 28	n = 109	n = 196
**Fluid intake (l)**	Mean (SD)	5.08 (2.47)	5.01 (2.20)	4.04 (1.52)	4.49 (2.01)
	Median [Q1, Q3]	4.50 [3.50,5.75]	4.43 [3.50,6.06]	4.00 [3.00,5.00]	4.00 [3.37,5.16]
		n = 59	n = 28	n = 108	n = 195
**Fluid output (l)**	Mean (SD)	1.10 (0.76)	1.39 (1.06)	0.91 (0.75)	1.04 (0.82)
	Median [Q1, Q3]	1.00 [0.58,1.33]	1.16 [0.65,1.86]	0.66 [0.40,1.30]	0.85 [0.46,1.35]
		n = 59	n = 28	n = 108	n = 195
**Total hospital length**	Mean (SD)	28.85 (23.23)	29.64 (25.37)	18.62 (12.35)	23.23 (18.96)
**of stay (days)**	Median [Q1, Q3]	20.00 [13.00,36.25]	24.00 [16.75,31.00]	14.00 [10.00,23.00]	17.00 [12.00,28.00]
		n = 60	n = 28	n = 112	n = 200
**ICU length of stay**	Mean (SD)	7.16 (15.99)	4.12 (12.14)	2.35 (6.91)	4.02 (11.21)
**(days)**	Median [Q1, Q3]	1.00 [1.00,3.00]	1.00 [1.00,2.75]	0.00 [0.00,1.00]	1.00 [0.00,2.00]
		n = 58	n = 26	n = 111	n = 195
**Clavien-Dindo**	I	18 (30.0%)	1 (3.6%)	44 (39.3%)	63 (31.5%)
	II	9 (15.0%)	2 (7.1%)	23 (20.5%)	34 (17.0%)
	IIIa	17 (28.3%)	18 (64.3%)	24 (21.4%)	59 (29.5)
	IIIb	3 (5.0%)	2 (7.1%)	13 (11.6%)	18 (9.0%)
	IVa	7 (11.7%)	1 (3.6%)	4 (3.6%)	12 (6.0%)
	IVb	1 (1.7%)	2 (7.1%)	0 (0.0%)	3 (1.5%)
	V	5 (8.3%)	2 (7.1%)	4 (3.6%)	11 (5.5%)
		n = 60	n = 28	n = 112	n = 200
**Clavien-Dindo**	Non-severe (< III)	27 (45.0%)	3 (10.7%)	67 (59.8%)	97 (48.5%)
**classification**	Severe (≥ III)	33 (55.0%)	25 (89.3%)	45 (40.2%)	103 (51.5%)
		n = 60	n = 28	n = 112	n = 200

### Regression analysis results

#### Colorectal surgery

**Fig. 1:**
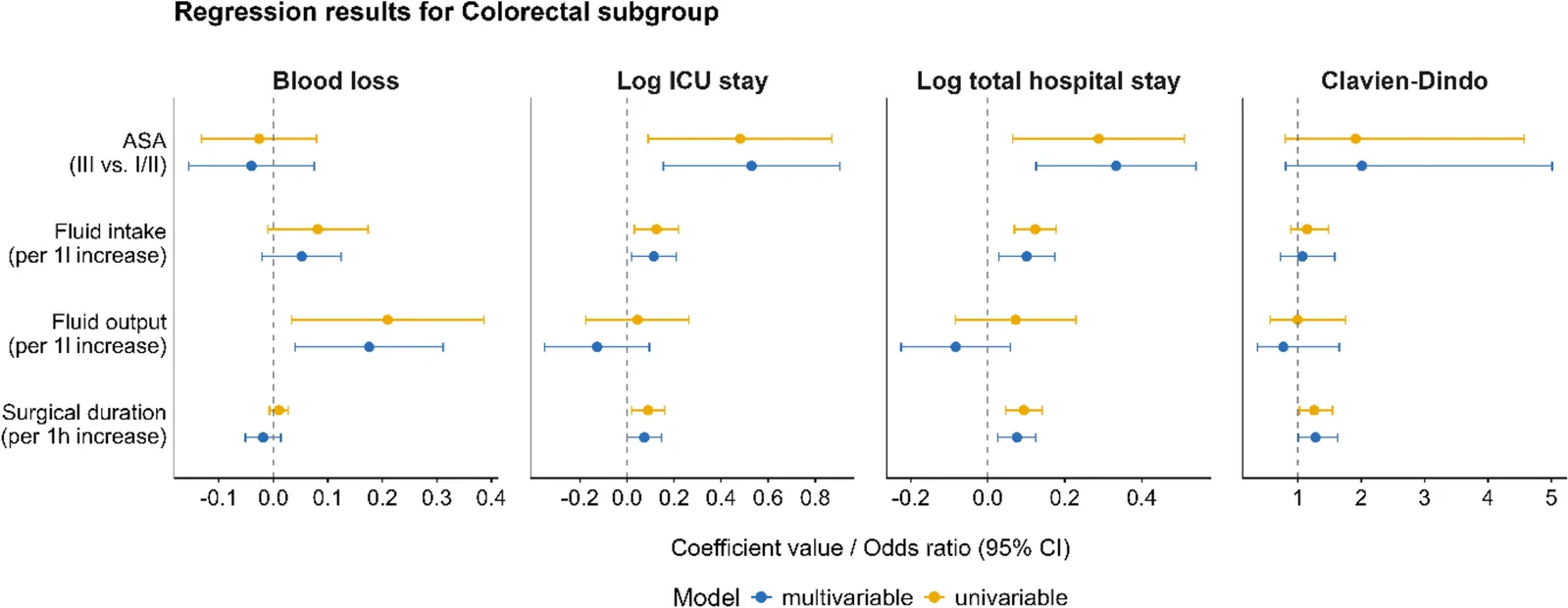
Forest plots of univariable and multivariable regression analyses for colorectal surgery. Linear regression models were used for the continuous outcomes blood loss, (log) ICU stay, and (log) total hospital stay, and logistic regression models for the dichotomized Clavien–Dindo classification. Regression coefficients and odds ratios are displayed as dots with corresponding 95% confidence intervals. The dotted vertical line indicates the null effect (0 for linear regression and 1 for logistic regression)

Figure 1 and Table S1 summarize the results of the regression analyses for the colorectal surgeries. In univariable analyses, ASA class III was associated with a longer total hospital stay compared to ASA class I/II, corresponding to an estimated 34% increase in hospital length of stay (β = 0.29, 95% CI 0.06–0.51; exp(0.29) = 1.34). Higher ASA class was further associated with longer ICU length of stay (β = 0.48, 95% CI 0.09–0.87; exp(0.48) = 1.62). No associations between ASA and Clavien–Dindo classification could be observed in our analysis.

Higher fluid intake was associated with prolonged total hospital stay in univariable models. Specifically, each liter increase in fluid intake was associated with an 13% increase in total hospital stay days (β = 0.12, 95% CI 0.07–0.18; exp(0.12) = 1.13). Naturally, fluid output was associated with increased blood loss (β = 0.21, 95% CI 0.03–0.39). Each additional hour of surgical duration was associated with a prolongation of hospital stay by 9% (β = 0.09, 95% CI 0.05–0.14, exp(0.09) = 1.09) and a 26% increase in the odds of severe complications according to the Clavien–Dindo classification (OR = 1.26, 95% CI 1.03–1.54).

After multivariable adjustment, the same variables remained statistically significant, with attenuated but directionally consistent estimates (Table S1). Notably, the association between ASA status and total hospital stay strengthened after adjustment for other covariates (β = 0.33, 95% CI 0.13–0.54; exp(0.33) = 1.39). This indicates that an increase from ASA status I/II to III was associated with a 39% increase in total hospital stay suggesting that ASA status captures independent risk beyond the other covariates included in the model.

#### Gastrointestinal (GI) surgery

**Fig. 2:**
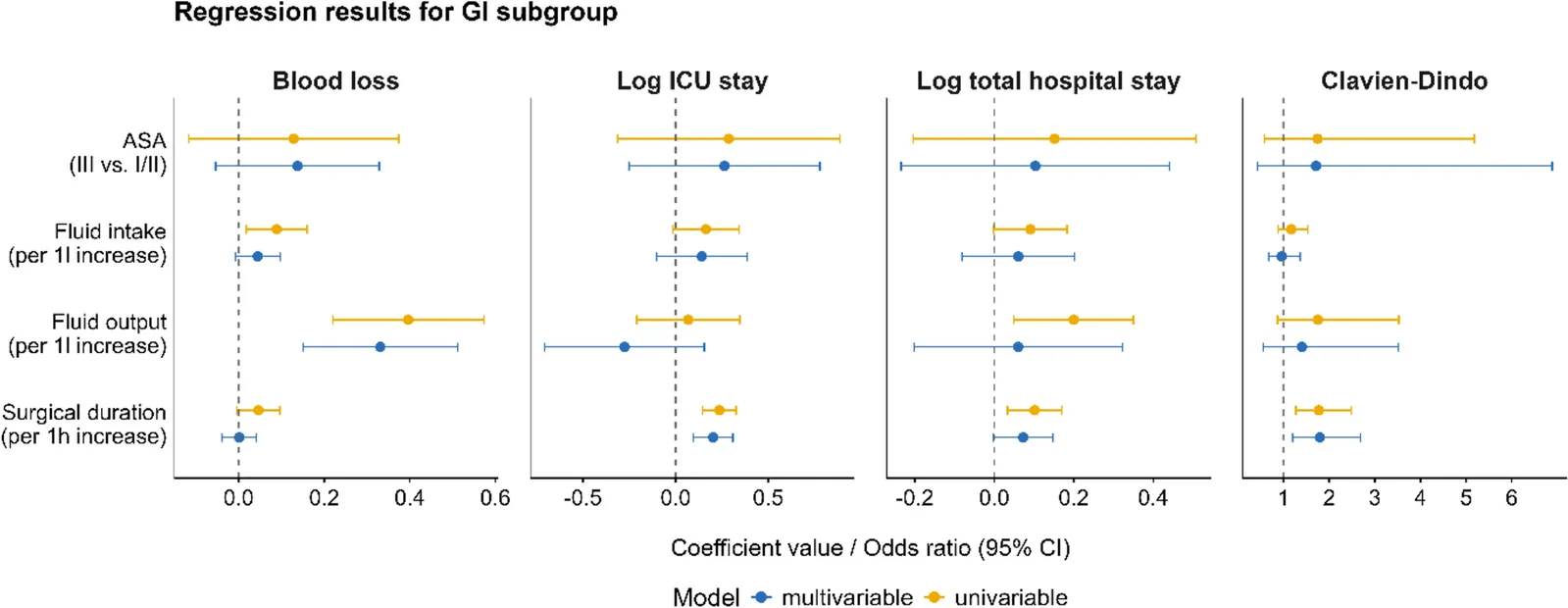
Forest plots of univariable and multivariable regression analyses for gastrointestinal surgery. Linear regression models were used for the continuous outcomes blood loss, (log) ICU stay, and (log) total hospital stay, and logistic regression models for the dichotomized Clavien–Dindo classification. Regression coefficients and odds ratios are displayed as dots with corresponding 95% confidence intervals. The dotted vertical line indicates the null effect (0 for linear regression and 1 for logistic regression)

Figure 2 and Table S2 present the results of the linear and logistic regression analyses for gastrointestinal surgeries. In univariable analyses, ASA status was not statistically significantly associated with any of the evaluated outcomes.

Higher intraoperative fluid intake was associated with increased blood loss in univariable analysis. Specifically, each one-liter increase in intraoperative fluid intake was associated with an increase in blood loss of 0.09 L (β = 0.09, 95% CI 0.02–0.16). In univariable analyses, fluid output was associated with blood loss, with each one-liter increase corresponding to an average increase of 0.40 L (β = 0.40, 95% CI 0.22–0.57). Longer surgical duration was associated with higher odds of severe complications according to the Clavien–Dindo classification, with each additional hour of operative time increasing the odds by approximately 77% (OR = 1.77, 95% CI 1.28–2.46).

Results from multivariable analyses were largely consistent with the univariable findings. The association between surgical duration and severe Clavien–Dindo complications remained significant in multivariable analysis. Additionally, fluid output showed a negative association with ICU stay, with each one-liter increase associated with a reduction of 24% (β = −0.28, 95% CI −0.71—0.15; exp(− 0.28) = 0.76). However, this effect did not reach statistical significance.

#### Hepatopancreaticobiliary (HPB) surgery

**Fig. 3:**
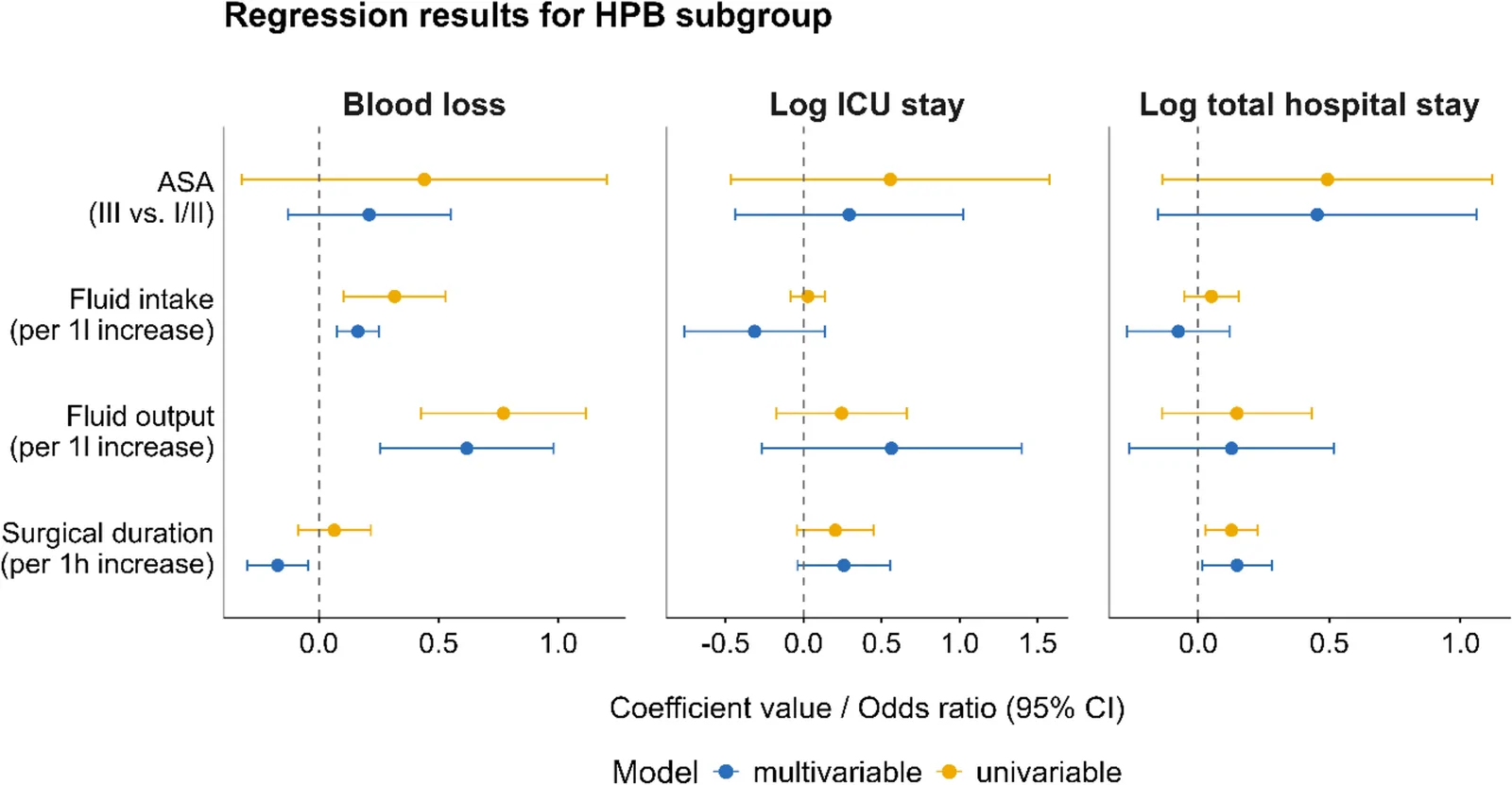
Forest plots of univariable and multivariable regression analyses for hepatobiliary surgery. Linear regression models were used for the continuous outcomes blood loss, (log) ICU stay, and (log) total hospital stay, and logistic regression models for the dichotomized Clavien–Dindo classification. Regression coefficients and odds ratios are displayed as dots with corresponding 95% confidence intervals. The dotted vertical line indicates the null effect (0 for linear regression and 1 for logistic regression). Fluid intake and output were analyzed as separate covariates, as the same net fluid balance can result from different combinations of fluid intake and output and may therefore mask potentially distinct associations of the two components with clinical outcomes. As a sensitivity analysis, net fluid balance (fluid intake minus fluid output) was additionally modeled as a single covariate. The results of this sensitivity analysis are presented in Supplementary Tables S4, S5 and S6 and were broadly consistent with the findings of the primary analysis

Figure 3 and Table S3 present the results of the regression analyses for hepatopancreaticobiliary surgeries. In univariable analyses, higher fluid intake (β = 0.32, 95% CI 0.10–0.53) and higher fluid output (β = 0.77, 95% CI 0.43–1.12) were both positively associated with increased blood loss, with each one-liter increase in intake or output corresponding to an average increase of 0.32 and 0.77 L of blood loss, respectively. Surgical duration was associated with a longer hospital length of stay, with each additional hour of surgery associated with a 14% increase in hospital length of stay (β = 0.13, 95% CI 0.03–0.23; exp(0.13) = 1.14).

Overall, the multivariable analyses yielded broadly similar findings. In addition, longer surgical duration was significantly associated with reduced blood loss (β = −0.17, 95% CI −0.30—−0.05).

## Discussion

This retrospective analysis of 200 patients undergoing robotic-assisted surgery across three major surgical specialties provides exploratory insights into important associations between anesthesiological parameters and perioperative outcomes. Our findings emphasize the association of surgical duration and fluid management with complication rates, ICU utilization, and hospital length of stay; importantly, analyses were conducted separately within each surgical category to account for the inherent procedural differences.

### Surgical duration and outcomes

One of the most consistent findings across our cohort was the association between prolonged surgical duration and adverse outcomes. In both GI and colorectal procedures, longer operative times were independently associated with severe complications (Clavien-Dindo grade ≥III), even after adjusting for patient-related factors and fluid management variables. This relationship aligns with previous literature demonstrating that prolonged surgery increases exposure to anesthetic agents, hypothermia risk, immunosuppression, and inflammatory responses [4]. The association could also reflect the complexity of the procedure which has been shown to be linked to outcome [9].

The mechanism underlying this association is likely multifactorial. Extended procedure times in robotic surgery may result from a range of factors including technical challenges, surgeon experience, or inherent case complexity. From an anesthesiological perspective, prolonged procedures increase the cumulative exposure to anesthetics and neuromuscular blocking agents, potentially affecting postoperative recovery. Additionally, extended periods in steep Trendelenburg position and pneumoperitoneum may have a compounding effect on hemodynamic and respiratory challenges. Since this is a retrospective analysis, one must also consider the inability to distinguish between cause and effect in this context, suggesting caution in the interpretation of these findings.

In HPB surgery, we observed a negative association between surgical duration and blood loss in multivariable analysis. This counterintuitive finding may reflect the fact that more experienced surgeons complete complex hepatopancreaticobiliary procedures more quickly while achieving superior hemostasis, or that cases with significant intraoperative bleeding may be expedited or converted more rapidly. Given the small sample size relative to the number of predictors examined in this subgroup, the HPB multivariable models are particularly susceptible to overfitting and unstable effect estimates; these findings should therefore be regarded as exploratory only and are not intended to support firm clinical inference.

### Fluid management and clinical outcomes

In our study, we observed associations between perioperative fluid management and multiple outcome parameters, with patterns varying across surgical specialties. In colorectal surgery, fluid intake was positively associated with both ICU length of stay and total hospital length of stay. These findings support the growing body of evidence favoring goal-directed or restrictive fluid therapy approaches, particularly in major abdominal surgery [10].

The physiological rationale for limiting excessive fluid administration includes prevention of tissue edema, preservation of endothelial glycocalyx integrity, and avoidance of complications such as anastomotic leak, ileus, and pulmonary complications. However, this does not appear to apply to the GI cohort where no association could be found. This is in line with previous results from Torabi et al. who could not show an association between fluid volumes and major complications in robot assisted esophagectomy [7].

We did not observe an association between fluid administration and complications in colorectal surgery, while a higher fluid intake was associated with an increased length of hospital stay. The relationship between fluid balance, perioperative complications and length of stay in colorectal surgery has been reported on many occasions [11]. To address this issue, a “zero balance” approach has been proposed as a pragmatic tool for a restrictive fluid regimen [12]. However, currently, a restrictive, but slightly positive fluid balance is recommended [10]. A restrictive regimen for robotic colorectal surgery has shown to be beneficial concerning the surgical field and length of ICU stay [5].

The observed association between fluid output (which includes blood loss and other fluid losses) and measured blood loss across all surgical groups serves as a validation of our data collection and analysis methods. However, the association between fluid output and ICU length of stay in GI surgery (with a negative association in multivariable analysis) requires careful interpretation and may reflect complex interactions between fluid resuscitation, blood loss, and critical illness severity.

### Complications and the Clavien-Dindo classification

The dichotomization of complications into non-severe (grades I-II) and severe (grades III-V) provided meaningful clinical insights. Within the HPB group, severe complications requiring intervention occurred in 89.3% of patients, consistent with the inherent complexity and risk profile of pancreaticoduodenectomy and similar major hepatopancreaticobiliary procedures. It should be noted that many grade IIIa events in this cohort, such as percutaneous drain interventions for postoperative pancreatic fistula, are anticipated complications inherent to major HPB surgery and may not represent unexpected adverse events in this population. In GI and colorectal procedures, severe complication rates were 55.0% and 40.2%, respectively, reflecting the specific risk profiles of these procedure types.

The independent association between surgical duration and severe complications in GI and colorectal surgery persisted even after adjusting for ASA classification, suggesting that operative time itself—or factors correlated with it—represents a modifiable risk factor. These findings support efforts to optimize surgical efficiency through enhanced surgeon training, improved case selection during the learning curve phase, and multidisciplinary perioperative planning [9]. The described relationship may also represent the influence of surgical complexity which has been shown to influence postoperative complications [9].

## Limitations

Generally, the observed associations between fluid administration and adverse perioperative outcomes should be interpreted with caution. Given the retrospective observational design, these associations cannot be interpreted as causal effects. Our analysis did not account for all potential confounders, including specific surgical procedures within each category, surgeon experience with robotic techniques, use of enhanced recovery after surgery (ERAS) protocols, or specific anesthetic techniques (e.g. epidural analgesia, intravenous lidocaine, or total intravenous anesthesia).

In particular, confounding by indication may have contributed to the observed findings. More complex or prolonged surgical procedures may require greater intraoperative fluid administration while simultaneously being associated with a higher risk of postoperative complications and prolonged ICU or hospital stays. Although our multivariable analyses accounted for selected available covariates, residual and unmeasured confounding cannot be excluded. Therefore, the observed associations should be considered exploratory and hypothesis-generating rather than evidence of a detrimental effect of higher fluid administration itself.

Additionally, given the exploratory nature of this study and the relatively large number of statistical analyses performed, the risk of false-positive findings due to multiple testing should be acknowledged. We therefore do not interpret individual p-values as confirmatory evidence, and p-values are reported in the results tables for descriptive purposes only. No adjustment for multiple comparisons was applied because the analyses were primarily hypothesis-generating and aimed at identifying potential associations and providing preliminary effect estimates to inform future studies, rather than testing a predefined set of confirmatory hypotheses. Nevertheless, the possibility of chance findings cannot be excluded, and the reported associations should therefore be interpreted cautiously and require confirmation in future studies.

Finally, our single-center experience may limit external validity, as practice patterns, patient populations, and surgical techniques may differ across institutions.

### Clinical implications and future directions

Our findings have several important clinical implications despite these limitations. Real-time awareness of operative duration may prompt anesthesiologists to intensify monitoring and consider interventions to mitigate complication risk in prolonged procedures. Concerning fluid management protocols, our results may allow the interpretation that the implementation of goal-directed or restrictive fluid therapy approaches, particularly in colorectal surgery, may improve outcomes. Future prospective studies are required to evaluate specific fluid management strategies tailored to robotic surgery requirements and potentially patient characteristics. With regard to adequate requirements for postoperative monitoring, incorporating intraoperative parameters such as fluid requirements into risk prediction models may improve identification of patients that benefit particularly from ICU admission.

Future research should prospectively evaluate the impact of specific anesthetic management strategies on robotic surgery outcomes. Randomized controlled trials comparing restrictive versus liberal fluid therapy, or goal-directed approaches using advanced hemodynamic monitoring, would provide higher-level evidence. Additionally, investigation of enhanced recovery protocols specifically designed for robotic surgery may identify additional opportunities for outcome improvement.

## Conclusions

In this retrospective analysis, we explored associations between anesthesiological parameters—particularly surgical duration and fluid management—and perioperative outcomes in robotic surgery. Prolonged operative time was independently associated with severe complications in GI and colorectal procedures. These findings emphasize the importance of optimized anesthetic techniques, judicious fluid management, and efforts to maximize surgical efficiency. Tailored perioperative management strategies specific to each surgical specialty may improve outcomes and facilitate the safe expansion of robotic surgical techniques. Prospective studies are warranted to validate these associations and evaluate specific interventions targeting modifiable anesthesiological risk factors.

## Supplementary Information

Below is the link to the electronic supplementary material.

Supplementary file1

## Data Availability

Restrictions apply to the availability of the data used for this retrospective analysis, which were used under licence for the current study and so are not publicly available. The data are, however, available upon reasonable request from the corresponding author.
