# Supplementary material for: Anesthesiological considerations and complications in robotic surgery: a retrospective analysis
Source: J Robot Surg. 2026 Oct 12;20(1):1091. doi: 10.1007/s11701-026-04045-6 (PMC13654881; doi:10.1007/s11701-026-04045-6)
Supplement: Supplementary file 1 [file 11701_2026_4045_MOESM1_ESM.docx]

**Supplementary Material**

**Table S1**

**Colorectal surgery**

Supplementary Table S1 Results of univariable and multivariable linear and logistic regression models on the different outcomes for colorectal surgeries. (Q1, Q3) is the first and third quartile and (95% CI) denotes the 95% confidence interval. N denotes the total number of observations per group and n denotes the number of valid observations. R^2^ denotes the R squared value of each univariable and the multivariable linear regression model and for the logistic regression models the AIC is mentioned.

| **Blood loss (l)** | **Univariable** | | | | | **Multivariable (R² = 0.31)** | | | |
| --- | --- | --- | --- | --- | --- | --- | --- | --- | --- |
| **Risk factor** | **Median (Q1,Q3) / n (%)** | **N** | **Coefficient (95% CI)** | **p-value** | **R²** | **Median (Q1,Q3) / n (%)** | **N** | **Coefficient (95% CI)** | **p-value** |
| Fluid intake (l) | 4.00 (3.00, 5.00) | 108 | 0.08 (-0.01, 0.17) | 0.082 | 0.16 | 4.00 (3.00, 5.00) | 107 | 0.05 (-0.02, 0.12) | 0.158 |
| Fluid output (l) | 0.66 (0.40, 1.30) | 108 | 0.21 (0.03, 0.39) | 0.020 | 0.25 | 0.65 (0.40, 1.30) | 107 | 0.18 (0.04, 0.31) | 0.012 |
| Surgical duration (h) | 7.15 (5.72, 8.80) | 107 | 0.01 (-0.01, 0.03) | 0.241 | <0.01 | 7.15 (5.72, 8.80) | 107 | -0.02 (-0.05, 0.01) | 0.258 |
| ASA 1/2 | 79 (72.5%) |  |  |  |  | 77 (72.0%) |  |  |  |
| ASA 3 | 30 (27.5%) | 109 | -0.03 (-0.13, 0.08) | 0.624 | <0.01 | 30 (28.0%) | 107 | -0.04 (-0.16, 0.08) | 0.491 |
| **Log ICU stay (log(days))** | **Univariable** | | | | | **Multivariable (R² = 0.17)** | | | |
| **Risk factor** | **Median (Q1,Q3) / n (%)** | **N** | **Coefficient (95% CI)** | **p-value** | **R²** | **Median (Q1,Q3) / n (%)** | **N** | **Coefficient (95% CI)** | **p-value** |
| Fluid intake (l) | 4.00 (3.00, 5.00) | 108 | 0.13 (0.03, 0.22) | 0.009 | 0.05 | 4.00 (3.00, 5.00) | 107 | 0.11 (0.02, 0.21) | 0.018 |
| Fluid output (l) | 0.66 (0.40, 1.30) | 108 | 0.04 (-0.17, 0.26) | 0.686 | <0.01 | 0.65 (0.40, 1.30) | 107 | -0.13 (-0.35, 0.10) | 0.260 |
| Surgical duration (h) | 7.19 (5.72, 8.80) | 108 | 0.09 (0.02, 0.16) | 0.013 | 0.06 | 7.15 (5.72, 8.80) | 107 | 0.07 (-0.00, 0.15) | 0.050 |
| ASA 1/2 | 81 (73.0%) |  |  |  |  | 77 (72.0%) |  |  |  |
| ASA 3 | 30 (27.0%) | 111 | 0.48 (0.09, 0.87) | 0.016 | 0.06 | 30 (28.0%) | 107 | 0.53 (0.15, 0.90) | 0.006 |
| **Log total hospital stay (log(days))** | **Univariable** | | | | | **Multivariable (R² = 0.28)** | | | |
| **Risk factor** | **Median (Q1,Q3) / n (%)** | **N** | **Coefficient (95% CI)** | **p-value** | **R²** | **Median (Q1,Q3) / n (%)** | **N** | **Coefficient (95% CI)** | **p-value** |
| Fluid intake (l) | 4.00 (3.00, 5.00) | 108 | 0.12 (0.07, 0.18) | <0.001 | 0.12 | 4.00 (3.00, 5.00) | 107 | 0.10 (0.03, 0.17) | 0.007 |
| Fluid output (l) | 0.66 (0.40, 1.30) | 108 | 0.07 (-0.08, 0.23) | 0.360 | 0.01 | 0.65 (0.40, 1.30) | 107 | -0.08 (-0.23, 0.06) | 0.250 |
| Surgical duration (h) | 7.19 (5.72, 8.80) | 108 | 0.09 (0.05, 0.14) | <0.001 | 0.15 | 7.15 (5.72, 8.80) | 107 | 0.08 (0.03, 0.12) | 0.003 |
| ASA 1/2 | 81 (72.3%) |  |  |  |  | 77 (72.0%) |  |  |  |
| ASA 3 | 31 (27.7%) | 112 | 0.29 (0.06, 0.51) | 0.012 | 0.06 | 30 (28.0%) | 107 | 0.33 (0.13, 0.54) | 0.002 |
| **Clavien-Dindo classification** | **Univariable** | | | | | **Multivariable (AIC = 144.23)** | | | |
| **Risk factor** | **Median (Q1,Q3) / n (%)** | **N** | **Odds ratio (95% CI)** | **p-value** | **AIC** | **Median (Q1,Q3) / n (%)** | **N** | **Odds ratio (95% CI)** | **p-value** |
| Fluid intake (l) | 4.00 (3.00, 5.00) | 108 | 1.14 (0.89, 1.48) | 0.303 | 148.14 | 4.00 (3.00, 5.00) | 107 | 1.07 (0.73, 1.57) | 0.727 |
| Fluid output (l) | 0.66 (0.40, 1.30) | 108 | 0.99 (0.57, 1.74) | 0.984 | 149.21 | 0.65 (0.40, 1.30) | 107 | 0.77 (0.36, 1.64) | 0.496 |
| Surgical duration (h) | 7.19 (5.72, 8.80) | 108 | 1.26 (1.03, 1.54) | 0.026 | 142.38 | 7.15 (5.72, 8.80) | 107 | 1.28 (1.01, 1.62) | 0.044 |
| ASA 1/2 | 81 (72.3%) |  |  |  |  | 77 (72.0%) |  |  |  |
| ASA 3 | 31 (27.7%) | 112 | 1.91 (0.81, 4.52) | 0.140 | 152.61 | 30 (28.0%) | 107 | 2.01 (0.82, 4.96) | 0.129 |

**Table S2**

**GI surgery**

Supplementary Table S2 Results of univariable and multivariable linear and logistic regression models on the different outcomes for gastrointestinal surgeries. (Q1, Q3) is the first and third quartile and (95% CI) denotes the 95% confidence interval. N denotes the total number of observations per group and n denotes the number of valid observations. R^2^ denotes the R squared value of each univariable and the multivariable linear regression model and for the logistic regression models the AIC is mentioned.

| **Blood loss (l)** | **Univariable** | | | | | **Multivariable (R² = 0.5)** | | | |
| --- | --- | --- | --- | --- | --- | --- | --- | --- | --- |
| **Risk factor** | **Median (Q1,Q3) / n (%)** | **N** | **Coefficient (95% CI)** | **p-value** | **R²** | **Median (Q1,Q3) / n (%)** | **N** | **Coefficient (95% CI)** | **p-value** |
| Fluid intake (l) | 4.50 (3.50, 5.75) | 59 | 0.09 (0.02, 0.16) | 0.015 | 0.23 | 4.50 (3.50, 5.75) | 59 | 0.04 (-0.01, 0.10) | 0.093 |
| Fluid output (l) | 1.00 (0.58, 1.33) | 59 | 0.40 (0.22, 0.57) | <0.001 | 0.43 | 1.00 (0.58, 1.33) | 59 | 0.33 (0.15, 0.51) | <0.001 |
| Surgical duration (h) | 8.00 (6.35, 9.79) | 59 | 0.05 (-0.00, 0.10) | 0.070 | 0.05 | 8.00 (6.35, 9.79) | 59 | 0.00 (-0.04, 0.04) | 0.945 |
| ASA 1/2 | 31 (52.5%) |  |  |  |  | 31 (52.5%) |  |  |  |
| ASA 3 | 28 (47.5%) | 59 | 0.13 (-0.12, 0.37) | 0.298 | 0.02 | 28 (47.5%) | 59 | 0.14 (-0.05, 0.33) | 0.155 |
| **Log ICU stay (log(days))** | **Univariable** | | | | | **Multivariable (R² = 0.32)** | | | |
| **Risk factor** | **Median (Q1,Q3) / n (%)** | **N** | **Coefficient (95% CI)** | **p-value** | **R²** | **Median (Q1,Q3) / n (%)** | **N** | **Coefficient (95% CI)** | **p-value** |
| Fluid intake (l) | 4.50 (3.50, 5.50) | 58 | 0.16 (-0.01, 0.34) | 0.070 | 0.13 | 4.50 (3.50, 5.50) | 58 | 0.14 (-0.10, 0.39) | 0.250 |
| Fluid output (l) | 1.00 (0.57, 1.31) | 58 | 0.07 (-0.21, 0.35) | 0.624 | <0.01 | 1.00 (0.57, 1.31) | 58 | -0.28 (-0.71, 0.15) | 0.205 |
| Surgical duration (h) | 8.00 (6.34, 9.71) | 58 | 0.24 (0.15, 0.33) | <0.001 | 0.23 | 8.00 (6.34, 9.71) | 58 | 0.20 (0.10, 0.31) | <0.001 |
| ASA 1/2 | 31 (53.4%) |  |  |  |  | 31 (53.4%) |  |  |  |
| ASA 3 | 27 (46.6%) | 58 | 0.29 (-0.31, 0.89) | 0.342 | 0.02 | 27 (46.6%) | 58 | 0.26 (-0.25, 0.78) | 0.309 |
| **Log total hospital stay (log(days))** | **Univariable** | | | | | **Multivariable (R² = 0.18)** | | | |
| **Risk factor** | **Median (Q1,Q3) / n (%)** | **N** | **Coefficient (95% CI)** | **p-value** | **R²** | **Median (Q1,Q3) / n (%)** | **N** | **Coefficient (95% CI)** | **p-value** |
| Fluid intake (l) | 4.50 (3.50, 5.75) | 59 | 0.09 (-0.00, 0.18) | 0.054 | 0.11 | 4.50 (3.50, 5.75) | 59 | 0.06 (-0.08, 0.20) | 0.396 |
| Fluid output (l) | 1.00 (0.58, 1.33) | 59 | 0.20 (0.05, 0.35) | 0.010 | 0.05 | 1.00 (0.58, 1.33) | 59 | 0.06 (-0.20, 0.32) | 0.648 |
| Surgical duration (h) | 8.00 (6.35, 9.79) | 59 | 0.10 (0.03, 0.17) | 0.004 | 0.12 | 8.00 (6.35, 9.79) | 59 | 0.07 (-0.00, 0.15) | 0.057 |
| ASA 1/2 | 31 (51.7%) |  |  |  |  | 31 (52.5%) |  |  |  |
| ASA 3 | 29 (48.3%) | 60 | 0.15 (-0.20, 0.51) | 0.396 | 0.01 | 28 (47.5%) | 59 | 0.10 (-0.23, 0.44) | 0.542 |
| **Clavien-Dindo classification** | **Univariable** | | | | | **Multivariable (AIC = 144.23)** | | | |
| **Risk factor** | **Median (Q1,Q3) / n (%)** | **N** | **Odds ratio (95% CI)** | **p-value** | **AIC** | **Median (Q1,Q3) / n (%)** | **N** | **Odds ratio (95% CI)** | **p-value** |
| Fluid intake (l) | 4.50 (3.50, 5.75) | 59 | 1.17 (0.89, 1.53) | 0.261 | 83.59 | 4.50 (3.50, 5.75) | 59 | 0.96 (0.68, 1.36) | 0.820 |
| Fluid output (l) | 1.00 (0.58, 1.33) | 59 | 1.75 (0.88, 3.48) | 0.107 | 83.09 | 1.00 (0.58, 1.33) | 59 | 1.40 (0.57, 3.45) | 0.461 |
| Surgical duration (h) | 8.00 (6.35, 9.79) | 59 | 1.77 (1.28, 2.46) | < 0.001 | 68.41 | 8.00 (6.35, 9.79) | 59 | 1.80 (1.21, 2.66) | 0.004 |
| ASA 1/2 | 31 (51.7%) |  |  |  |  | 31 (52.5%) |  |  |  |
| ASA 3 | 29 (48.3%) | 60 | 1.75 (0.60, 5.06) | 0.305 | 85.44 | 28 (47.5%) | 59 | 1.71 (0.44, 6.68) | 0.439 |

**Table S3**

**HPB surgery**

Supplementary Table S3 Results of univariable and multivariable linear and logistic regression models on the different outcomes for hepatobiliary surgeries. (Q1,Q3) is the first and third quartile and (95% CI) denotes the 95% confidence interval. N denotes the total number of observations per group and n denotes the number of valid observations. R^2^ denotes the R squared value of each univariable and the multivariable linear regression model.

| **Blood loss (l)** | **Univariable** | | | | | **Multivariable (R² = 0.5)** | | | |
| --- | --- | --- | --- | --- | --- | --- | --- | --- | --- |
| **Risk factor** | **Median (Q1,Q3) / n (%)** | **N** | **Coefficient (95% CI)** | **p-value** | **R²** | **Median (Q1,Q3) / n (%)** | **N** | **Coefficient (95% CI)** | **p-value** |
| Fluid intake (l) | 4.43 (3.50, 6.06) | 28 | 0.32 (0.10, 0.53) | 0.005 | 0.57 | 4.43 (3.50, 6.06) | 28 | 0.16 (0.08, 0.25) | <0.001 |
| Fluid output (l) | 1.16 (0.65, 1.86) | 28 | 0.77 (0.43, 1.12) | <0.001 | 0.79 | 1.16 (0.65, 1.86) | 28 | 0.62 (0.26, 0.98) | 0.002 |
| Surgical duration (h) | 7.65 (6.47, 8.36) | 28 | 0.06 (-0.09, 0.22) | 0.393 | 0.02 | 7.65 (6.47, 8.36) | 28 | -0.17 (-0.30, -0.05) | 0.010 |
| ASA 1/2 | 20 (71.4%) |  |  |  |  | 20 (71.4%) |  |  |  |
| ASA 3 | 8 (28.6%) | 28 | 0.44 (-0.32, 1.20) | 0.246 | 0.05 | 8 (28.6%) | 28 | 0.21 (-0.13, 0.55) | 0.214 |
| **Log ICU stay (log(days))** | **Univariable** | | | | | **Multivariable (R² = 0.32)** | | | |
| **Risk factor** | **Median (Q1,Q3) / n (%)** | **N** | **Coefficient (95% CI)** | **p-value** | **R²** | **Median (Q1,Q3) / n (%)** | **N** | **Coefficient (95% CI)** | **p-value** |
| Fluid intake (l) | 4.18 (3.50, 5.29) | 26 | 0.03 (-0.08, 0.14) | 0.605 | <0.01 | 4.18 (3.50, 5.29) | 26 | -0.31 (-0.76, 0.14) | 0.162 |
| Fluid output (l) | 1.01 (0.64, 1.56) | 26 | 0.24 (-0.17, 0.66) | 0.238 | 0.10 | 1.01 (0.64, 1.56) | 26 | 0.56 (-0.27, 1.40) | 0.173 |
| Surgical duration (h) | 7.55 (6.43, 8.20) | 26 | 0.20 (-0.04, 0.45) | 0.101 | 0.18 | 7.55 (6.43, 8.20) | 26 | 0.26 (-0.04, 0.56) | 0.083 |
| ASA 1/2 | 18 (69.2%) |  |  |  |  | 18 (69.2%) |  |  |  |
| ASA 3 | 8 (30.8%) | 26 | 0.56 (-0.46, 1.58) | 0.272 | 0.10 | 8 (30.8%) | 26 | 0.29 (-0.44, 1.02) | 0.415 |
| **Log total hospital stay (log(days))** | **Univariable** | | | | | **Multivariable (R² = 0.18)** | | | |
| **Risk factor** | **Median (Q1,Q3) / n (%)** | **N** | **Coefficient (95% CI)** | **p-value** | **R²** | **Median (Q1,Q3) / n (%)** | **N** | **Coefficient (95% CI)** | **p-value** |
| Fluid intake (l) | 4.43 (3.50, 6.06) | 28 | 0.05 (-0.05, 0.16) | 0.328 | 0.04 | 4.43 (3.50, 6.06) | 28 | -0.08 (-0.27, 0.12) | 0.429 |
| Fluid output (l) | 1.16 (0.65, 1.86) | 28 | 0.15 (-0.14, 0.43) | 0.298 | 0.07 | 1.16 (0.65, 1.86) | 28 | 0.13 (-0.26, 0.52) | 0.507 |
| Surgical duration (h) | 7.65 (6.47, 8.36) | 28 | 0.13 (0.03, 0.23) | 0.015 | 0.19 | 7.65 (6.47, 8.36) | 28 | 0.15 (0.02, 0.28) | 0.030 |
| ASA 1/2 | 20 (71.4%) |  |  |  |  | 20 (71.4%) |  |  |  |
| ASA 3 | 8 (28.6%) | 28 | 0.49 (-0.14, 1.12) | 0.119 | 0.15 | 8 (28.6%) | 28 | 0.45 (-0.15, 1.06) | 0.135 |

**Table S4**

**Colorectal surgery**

*Supplementary Table S4 Sensitivity analysis modeling net fluid balance (intake minus output) in place of fluid intake and fluid output separately. Results of univariable and multivariable linear and logistic regression models on the different outcomes for colorectal surgeries. (Q1, Q3) is the first and third quartile and (95% CI) denotes the 95% confidence interval. N denotes the total number of observations per group and n denotes the number of valid observations. R^2^ denotes the R squared value of each univariable and the multivariable linear regression model and for the logistic regression models the AIC is mentioned.*

| **Blood loss (l)** | **Univariable** | | | | | **Multivariable (R² = 0.5)** | | | |
| --- | --- | --- | --- | --- | --- | --- | --- | --- | --- |
| **Risk factor** | **Median (Q1,Q3) / n (%)** | **N** | **Coefficient (95% CI)** | **p-value** | **R²** | **Median (Q1,Q3) / n (%)** | **N** | **Coefficient (95% CI)** | **p-value** |
| Net fluid balance (l) | 3.20 (2.19, 3.85) | 108 | 0.04 (-0.03, 0.11) | 0.282 | 0.03 | 3.20 (2.17, 3.85) | 107 | 0.04 (-0.04, 0.11) | 0.337 |
| Surgical duration (h) | 7.15 (5.72, 8.80) | 107 | 0.01 (-0.01, 0.03) | 0.241 | <0.01 | 7.15 (5.72, 8.80) | 107 | 0.00 (-0.02, 0.03) | 0.752 |
| ASA 1/2 | 79 (72.5%) |  |  |  |  | 77 (72.0%) |  |  |  |
| ASA 3 | 30 (27.5%) | 109 | -0.03 (-0.13, 0.08) | 0.624 | <0.01 | 30 (28.0%) | 107 | -0.02 (-0.13, 0.08) | 0.660 |
| **Log ICU stay (log(days))** | **Univariable** | | | | | **Multivariable (R² = 0.32)** | | | |
| **Risk factor** | **Median (Q1,Q3) / n (%)** | **N** | **Coefficient (95% CI)** | **p-value** | **R²** | **Median (Q1,Q3) / n (%)** | **N** | **Coefficient (95% CI)** | **p-value** |
| Net fluid balance (l) | 3.20 (2.19, 3.85) | 108 | 0.15 (0.05, 0.24) | 0.003 | 0.06 | 3.20 (2.17, 3.85) | 107 | 0.12 (0.03, 0.20) | 0.011 |
| Surgical duration (h) | 7.19 (5.72, 8.80) | 108 | 0.09 (0.02, 0.16) | 0.013 | 0.06 | 7.15 (5.72, 8.80) | 107 | 0.07 (0.01, 0.14) | 0.034 |
| ASA 1/2 | 81 (73.0%) |  |  |  |  | 77 (72.0%) |  |  |  |
| ASA 3 | 30 (27.0%) | 111 | 0.48 (0.09, 0.87) | 0.016 | 0.06 | 30 (28.0%) | 107 | 0.53 (0.16, 0.90) | 0.006 |
| **Log total hospital stay (log(days))** | **Univariable** | | | | | **Multivariable (R² = 0.18)** | | | |
| **Risk factor** | **Median (Q1,Q3) / n (%)** | **N** | **Coefficient (95% CI)** | **p-value** | **R²** | **Median (Q1,Q3) / n (%)** | **N** | **Coefficient (95% CI)** | **p-value** |
| Net fluid balance (l) | 3.20 (2.19, 3.85) | 108 | 0.13 (0.07, 0.20) | <0.001 | 0.11 | 3.20 (2.17, 3.85) | 107 | 0.10 (0.03, 0.17) | 0.004 |
| Surgical duration (h) | 7.19 (5.72, 8.80) | 108 | 0.09 (0.05, 0.14) | <0.001 | 0.15 | 7.15 (5.72, 8.80) | 107 | 0.08 (0.03, 0.12) | 0.001 |
| ASA 1/2 | 81 (72.3%) |  |  |  |  | 77 (72.0%) |  |  |  |
| ASA 3 | 31 (27.7%) | 112 | 0.29 (0.06, 0.51) | 0.012 | 0.06 | 30 (28.0%) | 107 | 0.33 (0.13, 0.54) | 0.002 |
| **Clavien-Dindo classification** | **Univariable** | | | | | **Multivariable (AIC = 144.23)** | | | |
| **Risk factor** | **Median (Q1,Q3) / n (%)** | **N** | **Odds ratio (95% CI)** | **p-value** | **AIC** | **Median (Q1,Q3) / n (%)** | **N** | **Odds ratio (95% CI)** | **p-value** |
| Net fluid balance (l) | 3.20 (2.19, 3.85) | 108 | 1.19 (0.87, 1.63) | 0.272 | 147.81 | 3.20 (2.17, 3.85) | 107 | 1.08 (0.77, 1.54) | 0.648 |
| Surgical duration (h) | 7.19 (5.72, 8.80) | 108 | 1.26 (1.03, 1.54) | 0.026 | 142.38 | 7.15 (5.72, 8.80) | 107 | 1.25 (1.00, 1.57) | 0.053 |
| ASA 1/2 | 81 (72.3%) |  |  |  |  | 77 (72.0%) |  |  |  |
| ASA 3 | 31 (27.7%) | 112 | 1.91 (0.81, 4.52) | 0.140 | 152.61 | 30 (28.0%) | 107 | 1.99 (0.81, 4.85) | 0.133 |

**Table S5**

**GI surgery**

*Supplementary Table S5 Sensitivity analysis modeling net fluid balance (intake minus output) in place of fluid intake and fluid output separately. Results of univariable and multivariable linear and logistic regression models on the different outcomes for gastrointestinal surgeries. (Q1, Q3) is the first and third quartile and (95% CI) denotes the 95% confidence interval. N denotes the total number of observations per group and n denotes the number of valid observations. R^2^ denotes the R squared value of each univariable and the multivariable linear regression model and for the logistic regression models the AIC is mentioned.*

| **Blood loss (l)** | **Univariable** | | | | | **Multivariable (R² = 0.5)** | | | |
| --- | --- | --- | --- | --- | --- | --- | --- | --- | --- |
| **Risk factor** | **Median (Q1,Q3) / n (%)** | **N** | **Coefficient (95% CI)** | **p-value** | **R²** | **Median (Q1,Q3) / n (%)** | **N** | **Coefficient (95% CI)** | **p-value** |
| Net fluid balance (l) | 3.55 (2.77, 4.67) | 59 | 0.06 (-0.02, 0.14) | 0.114 | 0.09 | 3.55 (2.77, 4.67) | 59 | 0.06 (-0.02, 0.13) | 0.130 |
| Surgical duration (h) | 8.00 (6.35, 9.79) | 59 | 0.05 (-0.00, 0.10) | 0.070 | 0.05 | 8.00 (6.35, 9.79) | 59 | 0.03 (-0.02, 0.08) | 0.252 |
| ASA 1/2 | 31 (52.5%) |  |  |  |  | 31 (52.5%) |  |  |  |
| ASA 3 | 28 (47.5%) | 59 | 0.13 (-0.12, 0.37) | 0.298 | 0.02 | 28 (47.5%) | 59 | 0.13 (-0.11, 0.37) | 0.292 |
| **Log ICU stay (log(days))** | **Univariable** | | | | | **Multivariable (R² = 0.32)** | | | |
| **Risk factor** | **Median (Q1,Q3) / n (%)** | **N** | **Coefficient (95% CI)** | **p-value** | **R²** | **Median (Q1,Q3) / n (%)** | **N** | **Coefficient (95% CI)** | **p-value** |
| Net fluid balance (l) | 3.52 (2.76, 4.66) | 58 | 0.19 (-0.03, 0.41) | 0.083 | 0.15 | 3.52 (2.76, 4.66) | 58 | 0.14 (-0.10, 0.38) | 0.260 |
| Surgical duration (h) | 8.00 (6.34, 9.71) | 58 | 0.24 (0.15, 0.33) | <0.001 | 0.23 | 8.00 (6.34, 9.71) | 58 | 0.19 (0.09, 0.30) | <0.001 |
| ASA 1/2 | 31 (53.4%) |  |  |  |  | 31 (53.4%) |  |  |  |
| ASA 3 | 27 (46.6%) | 58 | 0.29 (-0.31, 0.89) | 0.342 | 0.02 | 27 (46.6%) | 58 | 0.27 (-0.25, 0.78) | 0.303 |
| **Log total hospital stay (log(days))** | **Univariable** | | | | | **Multivariable (R² = 0.18)** | | | |
| **Risk factor** | **Median (Q1,Q3) / n (%)** | **N** | **Coefficient (95% CI)** | **p-value** | **R²** | **Median (Q1,Q3) / n (%)** | **N** | **Coefficient (95% CI)** | **p-value** |
| Net fluid balance (l) | 3.55 (2.77, 4.67) | 59 | 0.09 (-0.03, 0.21) | 0.146 | 0.08 | 3.55 (2.77, 4.67) | 59 | 0.06 (-0.07, 0.20) | 0.347 |
| Surgical duration (h) | 8.00 (6.35, 9.79) | 59 | 0.10 (0.03, 0.17) | 0.004 | 0.12 | 8.00 (6.35, 9.79) | 59 | 0.08 (0.01, 0.16) | 0.035 |
| ASA 1/2 | 31 (51.7%) |  |  |  |  | 31 (52.5%) |  |  |  |
| ASA 3 | 29 (48.3%) | 60 | 0.15 (-0.20, 0.51) | 0.396 | 0.01 | 28 (47.5%) | 59 | 0.10 (-0.24, 0.44) | 0.551 |
| **Clavien-Dindo classification** | **Univariable** | | | | | **Multivariable (AIC = 144.23)** | | | |
| **Risk factor** | **Median (Q1,Q3) / n (%)** | **N** | **Odds ratio (95% CI)** | **p-value** | **AIC** | **Median (Q1,Q3) / n (%)** | **N** | **Odds ratio (95% CI)** | **p-value** |
| Net fluid balance (l) | 3.55 (2.77, 4.67) | 59 | 1.12 (0.84, 1.51) | 0.430 | 84.47 | 3.55 (2.77, 4.67) | 59 | 0.97 (0.70, 1.33) | 0.830 |
| Surgical duration (h) | 8.00 (6.35, 9.79) | 59 | 1.77 (1.28, 2.46) | < 0.001 | 68.41 | 8.00 (6.35, 9.79) | 59 | 1.82 (1.24, 2.67) | 0.002 |
| ASA 1/2 | 31 (51.7%) |  |  |  |  | 31 (52.5%) |  |  |  |
| ASA 3 | 29 (48.3%) | 60 | 1.75 (0.60, 5.06) | 0.305 | 85.44 | 28 (47.5%) | 59 | 1.70 (0.45, 6.39) | 0.434 |

**Table S6**

**HPB surgery**

*Supplementary Table S6 Sensitivity analysis modeling net fluid balance (intake minus output) in place of fluid intake and fluid output separately. Results of univariable and multivariable linear and logistic regression models on the different outcomes for hepatobiliary surgeries. (Q1,Q3) is the first and third quartile and (95% CI) denotes the 95% confidence interval. N denotes the total number of observations per group and n denotes the number of valid observations. R^2^ denotes the R squared value of each univariable and the multivariable linear regression model.*

| **Blood loss (l)** | **Univariable** | | | | | **Multivariable (R² = 0.5)** | | | |
| --- | --- | --- | --- | --- | --- | --- | --- | --- | --- |
| **Risk factor** | **Median (Q1,Q3) / n (%)** | **N** | **Coefficient (95% CI)** | **p-value** | **R²** | **Median (Q1,Q3) / n (%)** | **N** | **Coefficient (95% CI)** | **p-value** |
| Net fluid balance (l) | 3.44 (2.42, 5.05) | 28 | 0.31 (0.00, 0.61) | 0.047 | 0.23 | 3.44 (2.42, 5.05) | 28 | 0.40 (-0.01, 0.82) | 0.057 |
| Surgical duration (h) | 7.65 (6.47, 8.36) | 28 | 0.06 (-0.09, 0.22) | 0.393 | 0.02 | 7.65 (6.47, 8.36) | 28 | -0.10 (-0.32, 0.13) | 0.383 |
| ASA 1/2 | 20 (71.4%) |  |  |  |  | 20 (71.4%) |  |  |  |
| ASA 3 | 8 (28.6%) | 28 | 0.44 (-0.32, 1.20) | 0.246 | 0.05 | 8 (28.6%) | 28 | 0.61 (-0.15, 1.36) | 0.110 |
| **Log ICU stay (log(days))** | **Univariable** | | | | | **Multivariable (R² = 0.32)** | | | |
| **Risk factor** | **Median (Q1,Q3) / n (%)** | **N** | **Coefficient (95% CI)** | **p-value** | **R²** | **Median (Q1,Q3) / n (%)** | **N** | **Coefficient (95% CI)** | **p-value** |
| Net fluid balance (l) | 3.23 (2.40, 4.47) | 26 | -0.08 (-0.35, 0.19) | 0.555 | 0.02 | 3.23 (2.40, 4.47) | 26 | -0.24 (-0.61, 0.14) | 0.201 |
| Surgical duration (h) | 7.55 (6.43, 8.20) | 26 | 0.20 (-0.04, 0.45) | 0.101 | 0.18 | 7.55 (6.43, 8.20) | 26 | 0.29 (-0.05, 0.63) | 0.092 |
| ASA 1/2 | 18 (69.2%) |  |  |  |  | 18 (69.2%) |  |  |  |
| ASA 3 | 8 (30.8%) | 26 | 0.56 (-0.46, 1.58) | 0.272 | 0.10 | 8 (30.8%) | 26 | 0.42 (-0.40, 1.24) | 0.304 |
| **Log total hospital stay (log(days))** | **Univariable** | | | | | **Multivariable (R² = 0.18)** | | | |
| **Risk factor** | **Median (Q1,Q3) / n (%)** | **N** | **Coefficient (95% CI)** | **p-value** | **R²** | **Median (Q1,Q3) / n (%)** | **N** | **Coefficient (95% CI)** | **p-value** |
| Net fluid balance (l) | 3.44 (2.42, 5.05) | 28 | 0.04 (-0.12, 0.19) | 0.643 | <0.01 | 3.44 (2.42, 5.05) | 28 | -0.06 (-0.23, 0.11) | 0.483 |
| Surgical duration (h) | 7.65 (6.47, 8.36) | 28 | 0.13 (0.03, 0.23) | 0.015 | 0.19 | 7.65 (6.47, 8.36) | 28 | 0.15 (0.03, 0.28) | 0.016 |
| ASA 1/2 | 20 (71.4%) |  |  |  |  | 20 (71.4%) |  |  |  |
| ASA 3 | 8 (28.6%) | 28 | 0.49 (-0.14, 1.12) | 0.119 | 0.15 | 8 (28.6%) | 28 | 0.48 (-0.08, 1.04) | 0.088 |
